# Ru@hyperbranched Polymer for Hydrogenation of Levulinic Acid to Gamma-Valerolactone: The Role of the Catalyst Support

**DOI:** 10.3390/ijms23020799

**Published:** 2022-01-12

**Authors:** Svetlana A. Sorokina, Stepan P. Mikhailov, Nina V. Kuchkina, Alexey V. Bykov, Alexander L. Vasiliev, Mariam G. Ezernitskaya, Andrey L. Golovin, Linda Zh. Nikoshvili, Mikhail G. Sulman, Zinaida B. Shifrina

**Affiliations:** 1 A.N. Nesmeyanov Institute of Organoelement Compounds, Russian Academy of Sciences, 28 Vavilov St., 119991 Moscow, Russia; n_firsova@yahoo.com (N.V.K.); ezernits@mail.ru (M.G.E.); 2 Department of Biotechnology and Chemistry, Tver State Technical University, 22 A. Nikitina St., 170026 Tver, Russia; science@science.tver.ru (S.P.M.); bykovav@yandex.ru (A.V.B.); nlinda@yandex.ru (L.Z.N.); sulman@online.tver.ru (M.G.S.); 3 Federal Scientific Research Centre, Shubnikov Institute of Crystallography, “Crystallography and Photonics” of Russian Academy of Sciences, Leninsky Prospect, 59, 119333 Moscow, Russia; a.vasiliev56@gmail.com (A.L.V.); glavny@inbox.ru (A.L.G.)

**Keywords:** levulinic acid, gamma-valerolactone, ruthenium nanoparticle, hyperbranched polymer, hydrogenation, acid functionalization, heterogeneous catalyst

## Abstract

Hydrogenation of levulinic acid (LA) obtained from cellulose biomass is a promising path for production of γ-valerolactone (GVL)—a component of biofuel. In this work, we developed Ru nanoparticle containing nanocomposites based on hyperbranched pyridylphenylene polymer, serving as multiligand and stabilizing matrix. The functionalization of the nanocomposite with sulfuric acid significantly enhances the activity of the catalyst in the selective hydrogenation of LA to GVL and allows the reaction to proceed under mild reaction conditions (100 °C, 2 MPa of H_2_) in water and low catalyst loading (0.016 mol.%) with a quantitative yield of GVL and selectivity up to 100%. The catalysts were successfully reused four times without a significant loss of activity. A comprehensive physicochemical characterization of the catalysts allowed us to assess structure-property relationships and to uncover an important role of the polymeric support in the efficient GVL synthesis.

## 1. Introduction

Levulinic acid (LA) is one of the substances that can be obtained from cellulosic biomass via acid hydrolysis [1]. It is an important compound in biofuel production because it serves as a universal building block for syntheses of liquid fuel components [2]. In particular, gamma-valerolactone (GVL) obtained in the selective LA hydrogenation is a versatile intermediate for production of fuel additives and chemicals [3,4,5]. LA hydrogenation to GVL is a catalytic process in which both homogeneous [6] and heterogeneous [7,8] catalysts are used. Research is mainly focused on hydrogenation of LA and its esters by molecular hydrogen in the presence of metal catalysts [9]. Despite the high activity of homogeneous catalysts, the application of supported metal catalysts in industrial processes is especially beneficial due to ease of product recovery and the possibility of catalyst reuse.

Among numerous heterogeneous catalysts developed for LA transformation such as supported catalysts based on Ru [10,11,12,13,14], Co [15], Pt [16], Pd [17], Cu [18,19], and Ni [20,21], and Ru-containing systems are shown to be the most active. Typically, catalytically active metal is deposited on different supports including activated carbon [12,22], metal oxides [10,11,18], silica [14,17,21], and polymers [23,24]. In LA hydrogenation, a strong influence of the support on the catalysis rate, conversion and selectivity has been observed [25,26,27]. For example, Ru deposited on carbon was shown to be less active than that deposited on silica or alumina supports, while titanium oxide is considered as one of the most promising catalytic supports [10,26]. As a rule, to achieve a complete conversion and a high GVL yield, elevated temperatures of 150–200 °C and a hydrogen pressure of 1–5 MPa are needed [2,5,14,16,27,28,29,30].

A positive influence of the support acidity on catalytic LA hydrogenation has been demonstrated [22,28,31,32]. Modification of ordered mesoporous carbon containing Ru nanoparticles (NPs) with phosphoric acid significantly enhanced the activity compared to that of the non-modified catalyst [22]. In another work [31], the introduction of SO_3_H groups in polyethersulfone supported Ru nanoparticles led to a considerable increase of the LA hydrogenation rate. Moreover, the catalysts containing acidic groups can be used under milder reaction conditions providing a reasonable GVL yield. A presence of Lewis acid centers on the carrier such as TiO_2_, ZSM-5, and zeolites-β also has a high promotional effect on catalysis in terms of activity and selectivity [25,28,33,34,35,36]. This effect is explained by a better dispersion and stabilization of Ru NPs due to interactions between Ru NPs and acidic centers of the support [25,28,35].

Various dendrimers have been employed as support for Ru NPs for the catalysts utilized in the LA hydrogenation. Such catalysts demonstrated exceptional catalytic properties outmatching those for the catalysts based on conventional supports. The application of PPI dendrimers for stabilization of Ru NPs resulted in hybrid catalysts providing 78% GVL yield at 120 °C and 3 MPa of H_2_ in 2 h [14]. The fabrication of the dendrimer encapsulated Ru nanoparticulate catalyst allowed 94% of the GVL yield at 150 °C and 1 MPa of H_2_ in 5 h [16]. The enhanced activity of dendrimer-based catalysts is explained by a superior dispersion of catalytic NPs and good accessibility of active sites for substrate molecules. However, the major drawback of such systems is the complexity of dendrimer syntheses due to a time-consuming and cost prohibitive step by step approach for the organic skeleton construction. 

The hyperbranched polymers are an attractive alternative to dendrimers since they preserve the branched dendritic structure, while their synthesis is performed in a one-pot procedure. Here, we employed aromatic hyperbranched pyridylphenylene polymers (PPP) [37] that can provide an effective stabilization of Ru NPs in polar and non-polar media and survive the harsh condition of the catalytic reaction if needed. Another advantage of such polymers is an easy functionalization of pyridine groups that allows for controlled tuning of the properties of the resulting nanocomposite. An open structure of rigid hyperbranched polymers also allows for easy accessibility of catalytic NPs by substrate, leading to the improved catalysts performance [38]. We have previously demonstrated the efficacy of this approach for syntheses of Pd and Ni-containing PPP based catalysts for different types of organic reactions [38,39,40].

In this work, two Ru-containing nanoparticulate catalysts stabilized by PPP were designed using two different approaches. In one approach, thermal decomposition of Ru(acac)_3_ in the presence of PPP (Ru-PPP) was employed, while in the other approach, post-functionalization of Ru-PPP with sulfuric acid in mild conditions was used (Ru-PPP-S). Both catalysts were tested in LA hydrogenation to GVL in aqueous media and the influence of their composition and structure on catalytic performance was established.

## 2. Results and Discussion

**Figure 1:**
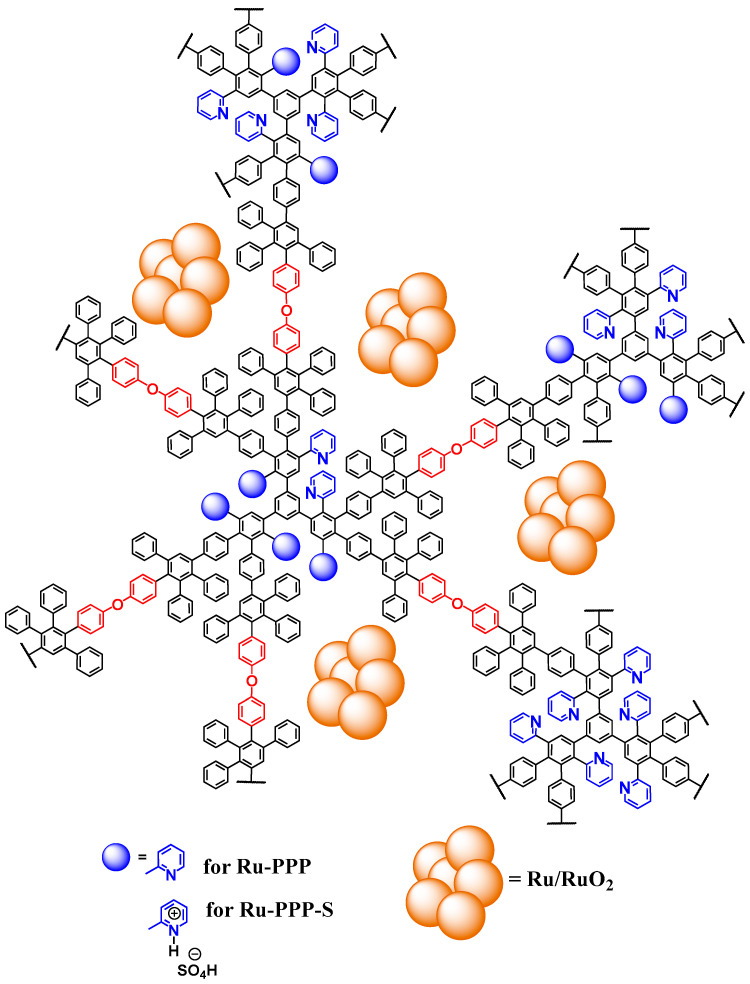
Schematic representation of Ru-containing catalysts based on PPP.

To study the hydrogenation of LA to GVL, we developed two types of Ru-containing polymer-based catalysts. Ru NPs were obtained by thermal decomposition of Ru(acac)_3_ in the reaction solution of pre-synthesized hyperbranched PPP [37] in benzyl ether at 285 °C in argon, giving the catalyst notated as Ru-PPP (Figure 1). Here, PPP serves as capping molecules for Ru NPs. As the LA hydrogenation is facilitated in the presence of acidic sites located in close proximity to the Ru species [22,32], we introduced acidic groups in Ru-PPP by a brief treatment with sulfuric acid (95–98%) at room temperature (sample Ru-PPP-S). This treatment should lead to protonation of polymer pyridine groups, resulting in quaternized pyridine moieties and HSO_4_^−^ counterions. Along with the acidic effect on the catalytic process efficiency, such a treatment increases hydrophilicity of the nanocomposite surface, thus facilitating the catalysis in water. Mild reaction conditions were used in the sulfuric acid treatment to avoid the possible Ru deactivation due to the sulfur presence [41,42,43].

### 2.1. Structure and Properties of Ru-PPP and Ru-PPP-S

**Figure 2:**
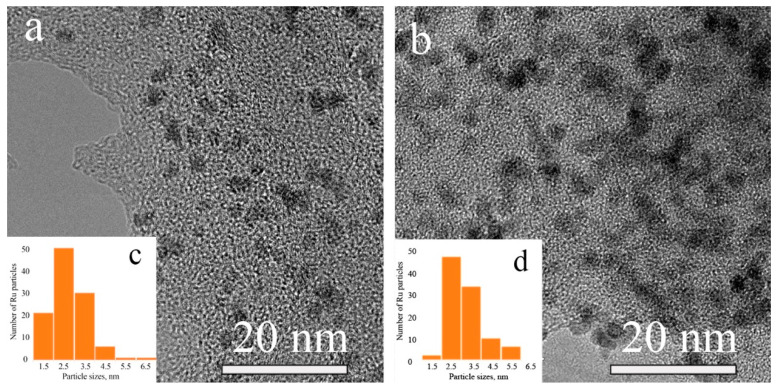
TEM images of Ru-PPP (**a**) and Ru-PPP-S (**b**) and Ru nanoparticles size distribution histograms for Ru-PPP (**c**) and Ru-PPP-S (**d**).

The size, size distribution and morphology of Ru-containing NPs were determined by transmission electron microscopy (TEM). The TEM images of Ru-PPP (Figure 2a) and Ru-PPP-S (Figure 2b) revealed that the Ru NPs are nearly spherical, measure 2.7 ± 0.7 nm and are uniformly distributed in the polymer matrix (Figure 2c,d). The comparison of the TEM image of Ru-PPP-S with that of Ru-PPP demonstrates that the sulfuric acid treatment leads to partial NP aggregation while the mean NPs size and morphology remain nearly the same with slight changes in the NP size distribution. This indicates that the post-treatment with sulfuric acid hardly affects the Ru NPs.

**Figure 3:**
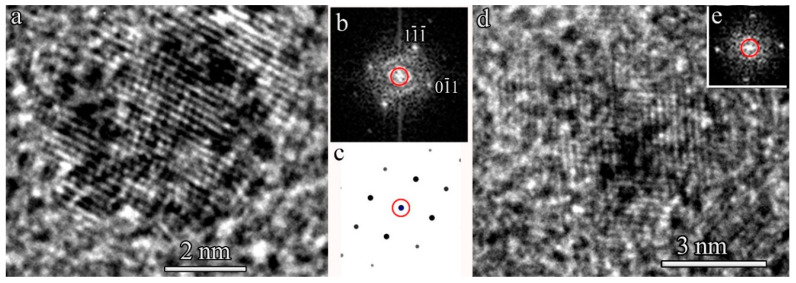
HRTEM images (**a**,**d**) and FFT patterns (**b**,**e**) of the Ru NPs in Ru-PPP (**a**,**b**) and Ru-PPP-S (**d**,**e**). Panel (**c**) shows a simulated electron diffraction pattern for hcp-Ru.

Fourier transform (FTT) patterns of HRTEM images with higher magnification (Figure 3) allowed us to evaluate the interplanar spacing in these NPs. The crystal lattice spacing was approximately 0.27 nm. The data for Ru-PPP-S show no change in the NP crystal structure after the sulfuric acid treatment.

To assess possible changes in the polymeric matrix upon the post-treatment, we employed the FTIR spectroscopy. FTIR spectra of Ru-PPP and Ru-PPP-S (Figure S1) demonstrate similar characteristic bands assigned to aromatic rings of PPP (see the Supporting Information for details). The treatment of the Ru-PPP composite with acid is accompanied by the emergence of a broad band at 3372 cm^−1^ and the band at 1376 cm^−1^ belonging to stretching and bending vibrations of N^+^-H groups, respectively. The absorption bands around 1219–1004 cm^−1^ are attributed to SO_2_ stretching and S-O-H bending vibrations of HSO_4_^−^ [44]. These data validate the presence of HSO_4_^−^ groups and quaternization of pyridine after the treatment of Ru-PPP with sulfuric acid.

**Figure 4:**
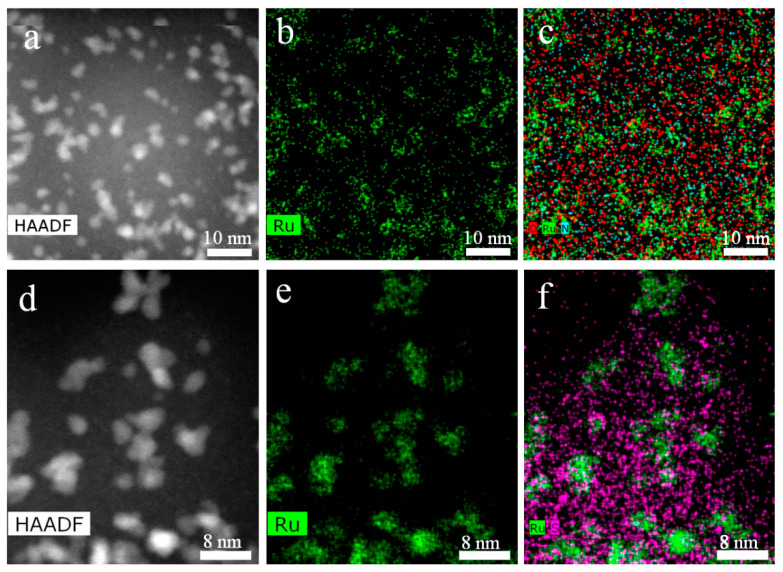
STEM dark-field image (**a**) and EDS maps for Ru (**b**), and Ru-O-N superpositions (**c**) for Ru-PPP and STEM dark-field image (**d**) and EDS maps for Ru (**e**), and Ru-S superpositions (**f**) for Ru-PPP-S.

STEM EDS mapping was utilized to evaluate the distribution of elements in the two catalysts. Figure 4 shows STEM dark-field images and EDS maps for Ru and the Ru-N-O superposition for Ru-PPP and Ru and the Ru-S superposition for Ru-PPP-S. Ru species spread all over the support with no visible Ru aggregates verifying a good stabilization of NPs with polymer in both catalysts. The Ru-N-O superposition (Figure 4c) for Ru-PPP confirms the presence of all species in the same location. S of Ru-PPP-S (Figure 4f) is evenly distributed in the sample. Ru-S superpositions revealed no obvious overlapping of S and Ru signals supporting the absence of S deposition on Ru NPs.

To validate that Ru NPs are metallic and crystalline even in the sample after the acidic treatment (Ru-PPP-S) X-ray diffraction (XRD) analysis has been used. The XRD pattern of Ru-PPP-S (Figure S2) shows a broad reflection at 43 two theta degrees, which corresponds to small Ru NPs [45,46]. From the deconvolution of this peak with further analysis of the (101) reflection using Sherrer’s equation, we found that Ru crystallites measure 2.7 nm, demonstrating that Ru NPs are single crystals.

**Figure 5:**
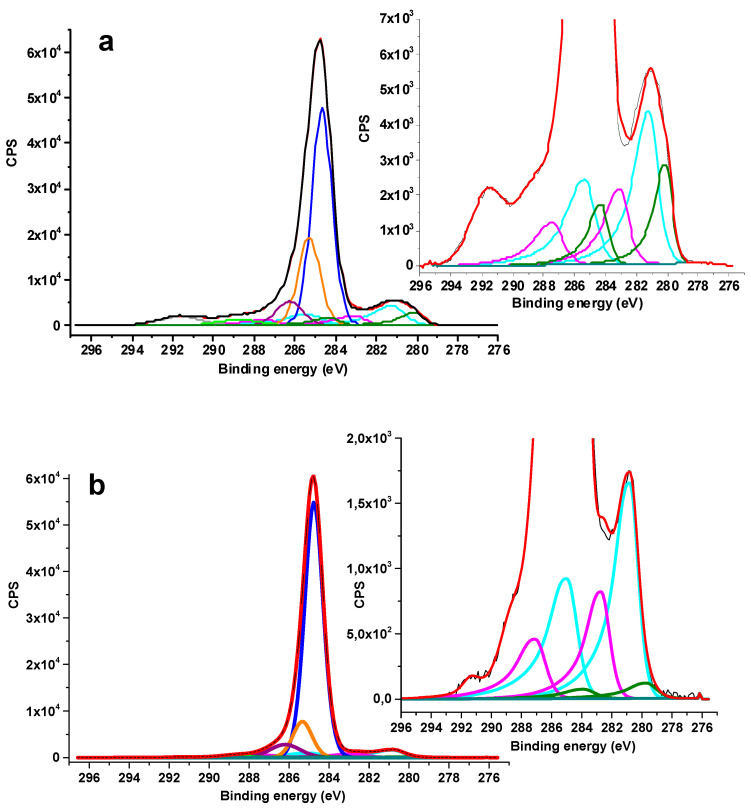
HR XPS C 1s and Ru 3d of Ru-PPP (**a**) and Ru-PPP-S (**b**). Black line is the experimental data, the blue line is for sp2 carbon, orange line is for C−N and adventitious sp3 carbon, the purple line is for C−O, the light green line is for COOH, the grey line is for π−π interactions in sp2, the olive lines are for Ru (0), the cyan lines are for RuO_2_, the magenta lines are for RuO_2_ satellite, the dark cyan line is for background and the red color is for the fitting curve. The scaled spectra depict the deconvolution of Ru spectrum without the lines corresponding to C 1s spectrum for more clarified representation. See Table S3 for the deconvolution data.

X-ray photoelectron spectroscopy (XPS) has been applied to assess the sample composition and the Ru oxidation state. The survey spectra presented in Figure S3 indicate that both samples contain C, O, N, and Ru, while the presence of S is observed for Ru-PPP-S. The elemental composition (Table S1) of Ru-PPP and Ru-PPP-S as well as detailed description of high resolution (HR) XPS C 1s spectrum along with the deconvolution parameters (Table S2) are given in the Supporting Information. Here, we will focus on deconvoluted Ru 3d peaks (Figure 5). The deconvolution of HR Ru 3d XPS spectra of Ru containing composites shows two types of species, Ru^0^ and Ru^4+^, at the different atomic ratio: 1:2.1 and 1:11.7, for Ru-PPP and Ru-PPP-S, respectively (see Table S3 for fitting parameters). Clearly, the sulfuric acid treatment under air leads to further oxidation of Ru^0^ on the Ru NP surface, resulting in the increase of the Ru^4+^ fraction in the surface layer. Considering that RuO_2_ is not detected by XRD and HRTEM, we believe that the oxide layer is very thin and/or amorphous.

The comparison of the HR XPS N 1s spectra before and after treatment with sulfuric acid shows the emergence of the peak at 401.7 eV, attributed to quaternized nitrogen [47] in Ru-PPP-S, while the peak at 399.3 eV corresponding to nitrogen bound to carbon is present in both samples (Figure S4a,b and Table S4 for fitting parameters). This demonstrates incomplete protonation of PPP pyridine moieties, which could be assigned to initial hydrophobicity of PPP and mild conditions of the treatment with sulfuric acid. The deconvolution of the HR S 2p3/2 spectrum of Ru-PPP-S (Figure S5, Table S5) revealed the presence of the S^6+^ oxidation state of sulfate anion.

Since the hydrogenation of LA to GVL generally proceeds at elevated temperatures and high pressures, the thermal stability is a crucial parameter for successful application of the catalyst in this process. To determine that, we used a thermal gravimetric analysis (TGA) (Figure S6) that demonstrated high thermal stability of both composites. The pronounced weight loss in the Ru-PPP-S sample above 250 °C can be attributed to the decomposition of sulfuric acid residue [48].

### 2.2. Catalytic Performance of Ru-PPP and Ru-PPP-S in Hydrogenation of LA to GVL

**Figure 6:**
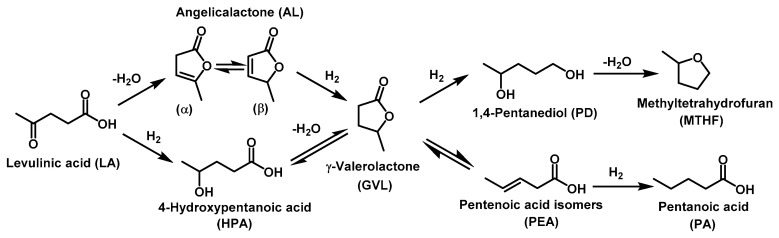
The possible pathways of hydrogenation of levulinic acid to γ-valerolactone.

The catalytic performance of Ru-PPP and Ru-PPP-S was investigated in the selective hydrogenation of LA to GVL. The hydrogenation of LA to GVL proceeds stepwise through the consecutive reactions of hydrogenation/dehydration with the formation of 4-hydroxypentanoic acid or angelica-lactones as intermediate products (Figure 6). The reaction pathway depends on the reaction conditions and the particular catalyst used [4,49,50]. In case of insufficient catalyst selectivity, different by-products such as angelica-lactones or overhydrogenated compounds (pentanoic acid (PA) or 2-methyltetrahydrofuran (MTHF)) are formed from further GVL transformations [49]. According to literature [9,50,51], utilization of Ru-containing catalysts and molecular H_2_ as reductant under mild reaction conditions (T < 150 °C, 1–5 MPa of H_2_) results in the 4-hydroxy-pentanoic acid-mediated path.

Generally, the hydrogenation of LA is performed in polar solvents, such as 1,4-dioxane, THF, alcohols, and water. The use of organic solvents usually provides the higher conversion of LA compared with the reaction in water [2,14,16,29,52]. Nevertheless, some authors point out the positive influence of water as a co-solvent on the reaction rate and the product yield [14,16,53]. Considering that, and being inspired by green chemistry benefits for environments, we used water as the sole reaction solvent. Moreover, in contrast to alcohols and cyclic esters, which may undergo side reactions of esterification or decomposition due to harsh reaction conditions, the use of water diminishes this effect and contributes to high reaction selectivity [25,27,28,54,55].

**Figure 7:**
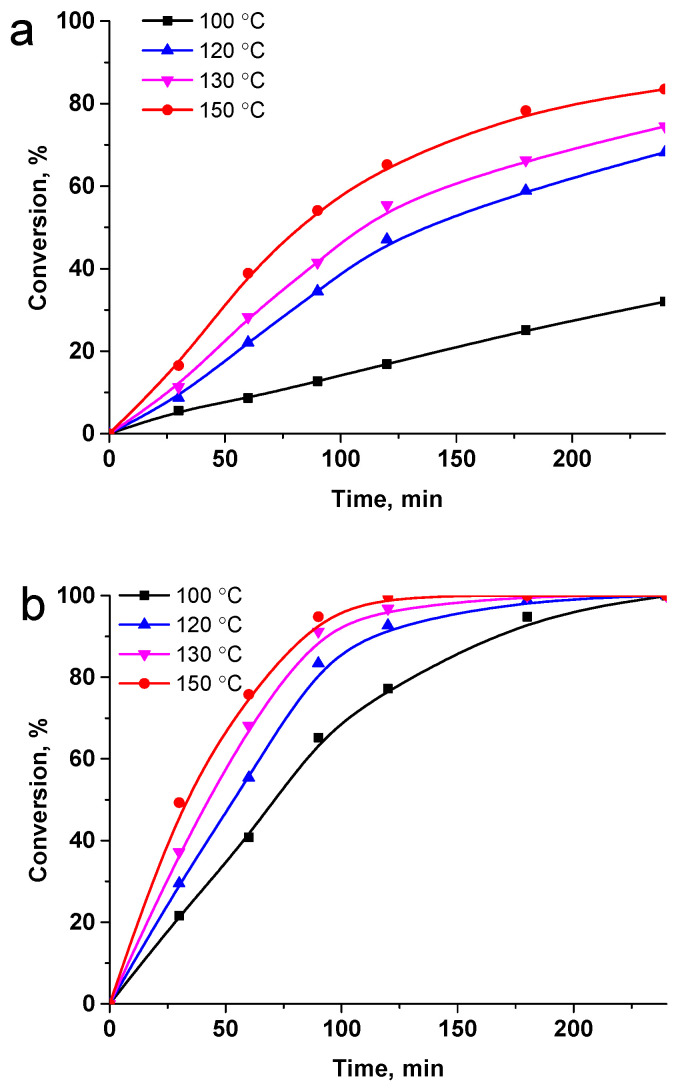
The effect of temperature on the hydrogenation of LA to GVL in the presence of Ru-PPP (**a**) and Ru-PPP-S (**b**) in the range of temperatures and under constant H_2_ pressure of 2 MPa. Reaction conditions: LA 1 g, solvent (H_2_O) 50 mL, catalyst loading 0.064 mol.% of Ru for Ru-PPP and 0.016 mol.% for Ru-PPP-S.

The catalytic performances of Ru-PPP and Ru-PPP-S were studied over the range of temperatures and pressures. Figure 7 shows the influence of temperature on LA conversion under the 2 MPa pressure of H_2_ for both catalysts. The data indicate that both catalysts are efficient in LA hydrogenation and the temperature increase leads to the anticipated increase in conversion. Ru-PPP-S appears to be more active, demonstrating a high conversion for 180 min even at 100 °C. The increase of the reaction temperature to 150 °C results in 94.8% conversion for 90 min. Ru-PPP shows much lower activity with only 32.0% conversion for 240 min at 100 °C. The increase in the temperature to 150 °C drastically improves the catalytic behavior of Ru-PPP, resulting in the conversion of 83.5% for 240 min. However, the complete conversion was still not observed under reaction conditions used (2 MPa of H_2_, 240 min).

**Figure 8:**
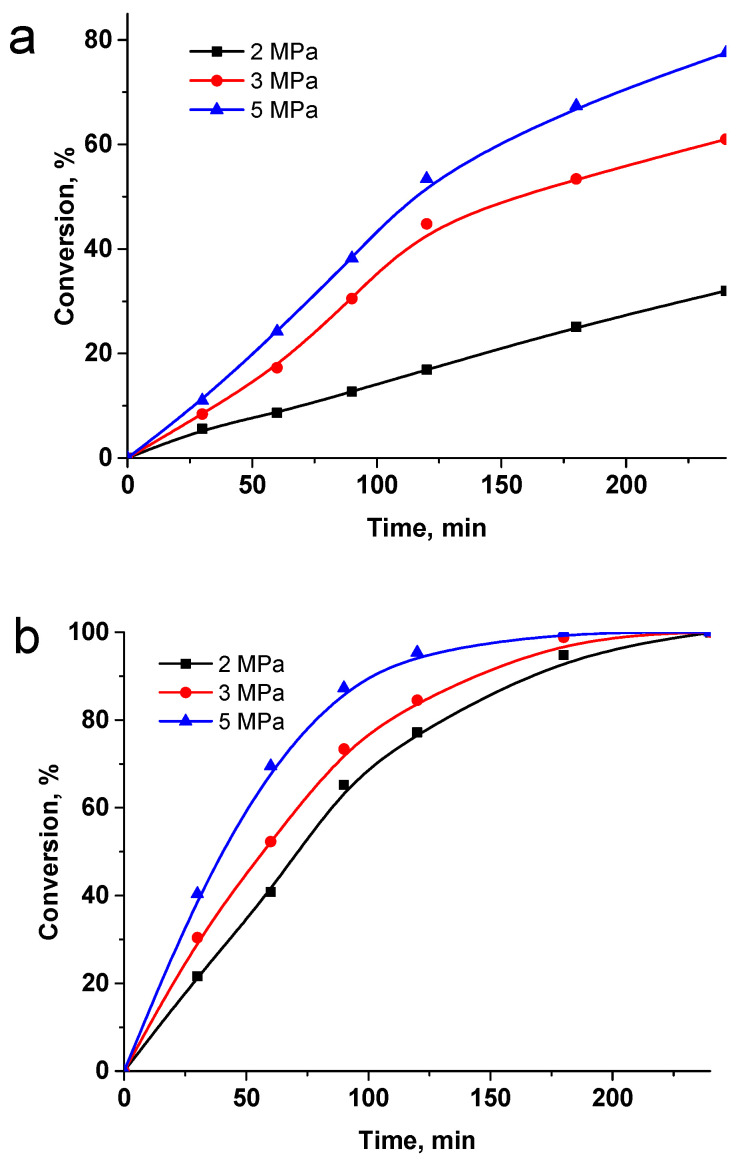
The effect of pressure on the hydrogenation of LA to GVL in the presence of Ru-PPP (**a**) and Ru-PPP-S (**b**) at 100 °C. Reaction conditions: LA 1 g, solvent 50 mL, catalyst loading 0.064 mol.% of Ru for Ru-PPP and 0.016 mol.% for Ru-PPP-S.

The effect of pressure was also investigated using 2, 3 and 5 MPa of H_2_ (Figure 8). A similar trend was observed: the pressure increase leads to the increase in conversion.

It should be noted that no intermediate products, such as angelica lactone, have been detected for both catalysts. It may indicate that the reaction proceeds through the formation of unstable 4-hydroxypentanoic acid, which undergoes rapid cyclization to GVL and is constituent with the literature data [9,50,51]. The high selectivity and yield of GVL along with the mild reaction conditions are characteristic for this route [4]. The use of Ru-PPP and Ru-PPP-S allows for quantitative yields of GVL within 4 h, but the conversion of LA differs dramatically. The data on catalytic performance of the catalysts are summarized in Table 1.

We believe that the improved catalytic behavior of Ru-PPP-S over Ru-PPP can be attributed to the presence of acidic groups in the catalytic nanocomposite. According to the literature data, acidic additives enhance the catalytic performance in LA hydrogenation [22,32]. For example, the acid functionalization of carbon support increased the catalytic activity by a factor of two in comparison with non-functionalized catalyst [22]. The addition of acidic co-catalyst, Amberlyst A70 or A15, to carbon-supported Ru catalyst increased the reaction rate and allowed the reaction to be performed under mild conditions [32]. This effect was attributed to promotion of the dehydration step in a cascade of LA hydrogenation reactions due to protonation of carbonyl group and fast water elimination.

In our case, the presence of HSO_4_^−^ ions in Ru-PPP-S in aqueous solution results in a release of protons in the swollen protonated hyperbranched PPP, which serves as a stabilizing medium to both Ru NPs and positively and negatively charged ions. Moreover, the quaternized pyridine molecules may contribute to enhanced catalytic activity because of the ion pair formation with levulinate anion in close proximity of the Ru NP surface. This facilitates the hydrogenation reaction due to the entrapment of reacting molecules in the PPP space. The similar effect was observed for PPI dendrimers [14] and N-doped carbon [56]. It is noteworthy that considering a limited thermal stability of Ru-PPP-S (see Figure S6 and discussion underneath), the use of more severe reaction conditions would be unwise since decomposition of acidic groups takes place. Moreover, the increase in temperature and pressure led to the selectivity decrease (Table 1). These results are in good agreement with the literature data: the acidic supports were shown to mediate the GVL ring-opening followed by its consecutive hydrogenation under harsh reaction conditions [22,27]. This leads to the decrease in the yield of target product.

We believe that minimization of material and energy resources is always preferable and should lead the catalyst development. Therefore, the excellent performance of Ru-PPP-S under mild reaction conditions (100 °C, 2 MPa) in water and low catalyst loading (0.016 mol.%) is highly beneficial for a green catalysis. The analysis of the literature in the field presented in Table 2 shows that similar catalytic results were obtained only under harsher conditions or longer reaction times or greater catalyst loadings.

**Figure 9:**
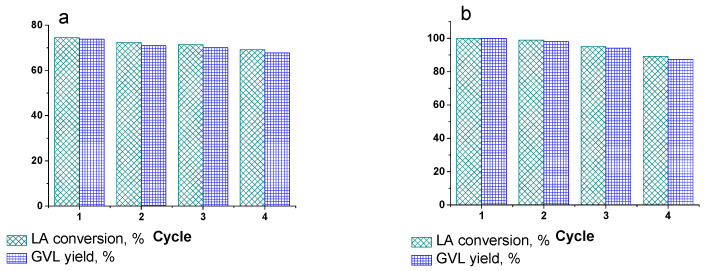
Recycling experiments for Ru-PPP (**a**) and Ru-PPP-S (**b**) in LA hydrogenation. Reaction conditions are: 130 °C, 2 MPa, 0.016 mol.% Ru, 4 h for Ru-PPP and 100 °C, 2 MPa, 0.064 mol.% Ru, 4 h for Ru-PPP-S.

To further test applicability of the catalysts developed for practical applications, we studied their recyclability in four consecutive catalytic runs. After the first LA hydrogenation, the catalyst was separated from the reaction mixture via centrifugation, thoroughly washed with water and used with the new portions of LA and solvent. The results of recycling are presented in Figure 9. Both catalysts preserve their activity during repeated use. The major drop of the GVL yield is observed upon the fourth cycle. Noticeably, Ru-PPP-S is characterized with the greater total loss of activity (percentage) in comparison with Ru-PPP. While for Ru-PPP, the GVL yield decreases by 6.1% after four catalytic cycles, the total loss of the GVL yield for Ru-PPP-S equals 12.6%. Nevertheless, the acid-modified Ru-PPP-S catalyst was still more active, giving 87% of GVL after four catalytic cycles.

To ensure the absence of possible Ru leaching, the ICP analyses of the supernatant after the first and second catalytic cycles were performed. The Ru amount in the supernatant was 400 and 600 ppb after the first and second catalytic runs, correspondingly, revealing the high stability of the catalysts.

It is established that the S-containing impurities can have a poisoning effect on Ru catalysts [61]. However, Ftouni et al. demonstrated a strong dependence of sulfur deactivation effect on the type of the support in the catalytic composite [62]. While Ru/C showed a significant deactivation in the presence of minor amounts of sulfuric acid in LA hydrogenation, Ru/ZrO_2_ preserved the activity and >99% selectivity. Moreover, the Ru/ZrO_2_ catalyst exhibited excellent stability in five recycling tests when the reaction was carried out in water. This effect was attributed to a scavenging capacity of ZrO_2_ support toward sulfur compounds. We propose that the PPP molecules may act similarly, forming a protective shield for the Ru active sites in case of Ru-PPP-S.

## 3. Materials and Methods

### 3.1. Materials

Ruthenium (III) acetylacetonate (Acros Organics (Geel, Belgium), 97%), sulfuric acid (Σtec (Moscow, Russia), 95–98%), and benzyl ether (Sigma-Aldrich (St. Louis, MO, USA), 98%) were used as received. Levulinic acid (≥98%) was purchased from Merck KGaA, Darmstadt, Germany. Gamma-valerolactone (ReagentPlus^®^ (Moscow, Russia), 99%) was purchased from Sigma-Aldrich and used as received. Acetone (99.5%) and chloroform (99.8%) were purchased from Component-reactive (Moscow, Russia) and used without purification. Ethanol (96%) was purchased from Chimmed (Moscow, Russia) and used as received. PPP were synthesized as described elsewhere [37]. According to size exclusion chromatography (SEC), average molecular weight of the polymer was 62,704 g/mol with the polydispersity coefficient 2.9.

### 3.2. Synthetic Procedures

#### 3.2.1. Preparation of the Ru-PPP

Synthesis of Ru-PPP in the presence of the PPP was carried out according to the following protocol [38]. In a typical procedure for the Ru-PPP synthesis, a three-neck round-bottom flask (with elongated necks) equipped with a magnetic stir bar, a reflux condenser, and two septa, one of which contained an inserted temperature probe protected with a glass shield and the other had a long needle for Ar inlet, was loaded with 0.398 g (1 mmol) of Ru(acac)_3_, 0.148 g (0.04 mmol) of the PPP, and 7 mL of benzyl ether. The flask was degassed by argon bubbling for 15 min under stirring at room temperature. Then, the temperature was raised to 60 °C at 10°/min and reaction was kept under stirring at this temperature for 30 min to allow solubilization. Then, the temperature was increased with a heating rate 10°/min until stabilizing around 283−285 °C (boiling) and reaction was kept at this temperature for 1 h. The flask was then removed from the heating mantle and allowed to cool to room temperature. The reaction solution was precipitated by ethanol then washed several times with ethanol and acetone until the supernatant was colorless, then dissolved in chloroform for storing in the refrigerator. Ru content (8.1%) was determined by X-ray fluorescence spectroscopy.

#### 3.2.2. Preparation of the Ru-PPP-S

Next, 84.4 mg of PPP-Ru and 5.2 mL of sulfuric acid (96%) were stirred at room temperature for 30 min. Then, the sample was filtrated through Schott filter and washed with distillated water. Sample was dried in vacuo at 70 °C before constant weight (elemental analysis found: S, 3.29%; Ru, 2.1%).

### 3.3. Catalytic Study

Hydrogenation of levulinic acid (LA) was carried out in Parr Series 5000 Multiple Reactor System (autoclave type reactor) at a stirring rate of 1500 rpm, at variation of such process parameters as temperature (100−150 °C) and hydrogen partial pressure (2−5 MPa). In a typical experiment, the sample of catalyst corresponding to 0.064 mol.% of Ru for Ru-PPP and 0.016 mol.% for Ru-PPP-S in reaction, 1 g of LA and 50 mL of solvent (distilled water) were placed into the reactor. Then, the reactor was sealed, purged with nitrogen (0.02 MPa) and heated up under mixing. Upon reaching the chosen temperature, nitrogen was replaced with hydrogen, pressure was adjusted, and the reaction was started (time “zero” for the reaction).

Samples of the reaction mixture were analyzed via GC (Kristallux 4000 M (Chromatec, Yoshkar-Ola, Russia)) equipped with FID and capillary column ZB-WAX (60 m × 0.53 mm i.d., 1 μm film thickness). Temperatures of detector and injector were 250 °C and 300 °C, respectively. Column temperature was programmed as follows: 150 °C (13 min) then heating up to 230 °C (30 °C/min) and then 230 °C for 7 min. Helium (30 mL/min) was used as a carrier gas. The concentrations of the reaction mixture components were calculated using an absolute calibration method using chemically pure components of reaction mixture.

Conversion of LA was defined as *X_LA_* (%) = (*C_LA_*_,0_ − *C_LA_*) × *C_LA_*_,0_^−1^ × 100, and selectivity with respect to GVL was given as *S_GVL_* (%) = *C_GVL_* × (*C_LA_*_,0_ − *C_LA_*)^−1^ × 100. The GVL yield was calculated as multiplication of conversion and selectivity values.

### 3.4. Recycling Experiment

After the completion of LA hydrogenation, the catalyst was centrifuged (6000 rpm, 15 min) and washed with water (500 mL). Then, it was dried till constant weight at 70 °C. It is noteworthy that several catalyst samples were collected from previous runs and average catalyst sample was taken for further run to keep all the reaction conditions unchanged including the catalyst weight. The subsequent catalytic experiment was carried according to standard procedure described above.

### 3.5. Characterization

Specimens for TEM, STEM, and EDXS studies were prepared by dipping the Lacey carbon film on the Cu grid into the vail with the Ru/polymer powder. The study of the samples by transmission electron microscopy (TEM), scanning transmission electron microscopy (STEM), electron diffraction (ED), and energy-dispersive X-ray (EDX) microanalysis was carried out in an Osiris TEM/STEM (Thermo Fisher Scientific, Waltham, MA, USA) equipped with a high angle annular dark field detector (HAADF) (Fischione, Corporate Circle Export, PA, USA) and an X-ray energy dispersive spectrometer Super X (ChemiSTEM, Bruker, Billerica, MA, USA) at an accelerating voltage of 200 kV. Image processing was performed using a Digital Micrograph (Gatan, Pleasanton, CA, USA) and TIA (ThermoFisher Scientific, Waltham, MA, USA) software. The dark field TEM images together with the high-angle annular dark-field scanning transmission electron microscopy (HAADF STEM) images were used for the determination of the particle size distribution. The particle size distribution histograms were obtained for approximately 100 particles.

XRD measurements were carried out in a Rigaku MiniFlex600 diffractometer (Rigaku Corporation, Japan) using SiKa radiation (40 kV, 15 mA, Ni-Kß filter) in the angular range 2θ = 20–80° with a scanning step of 0.02° and a speed of 0.5°/min. The size of the beam incident on the sample was set by horizontal and vertical slits—10 mm and 1.25°, respectively. Identification was performed with the PDXL software (Rigaku Corporation, Japan) using the ICDD PDF-2 database (2017).

X-ray photoelectron spectroscopy (XPS) data were obtained using monochromatic Al Kα radiation with Axis Ultra DLD (Kratos) spectrometer. All the data were acquired at X-ray power of 150 W. Survey spectra were recorded at an energy step of 1 eV with an analyzer pass energy 160 eV, and high resolution spectra were recorded at an energy step of 0.1 eV with an analyzer pass energy 40 eV. Samples were allowed to outgas for 180 min before analysis and were stable during the examination. The data analysis was performed by CasaXPS.

To obtain the Ru content from elemental analysis, X-ray fluorescence (XRF) measurements were performed with a Zeiss Jena VRA-30 spectrometer equipped with a Mo anode, a LiF200 crystal analyzer, and a SD detector. The time of data acquisition was held constant at 10 s. Analyses were based on the RuKα line, and a series of standards were prepared by mixing 1 g of polystyrene with 10–20 mg of standard compounds. Elemental analysis for C, H, N and S was carried out using Vario Microcube micro analyzer (Elementar).

Thermal gravimetric analysis (TGA) was performed on Shimadzu DTG-60H (Shimadzu GmbH, Kyoto, Japan) at a heating rate of 10°/min under an argon atmosphere.

FTIR spectra were recorded on a Vertex 70 V Fourier spectrometer (Bruker, Berlin, Germany) using a Pike ATR accessory with a diamond crystal (Nicolet, Waltham, MA, USA); the ATR spectra were averaged from 128 scans over a range of 4000–400 cm^−1^ with a resolution of 4 cm^−1^. All necessary corrections were done using an Omnic 8 program package (Nicolet, USA).

The inductively coupled plasma optical emission spectroscopy (ICPOES) analyses were carried out using an Agilent ICP-OES5110 apparatus (Agilent, Santa Clara, CA, USA).

## 4. Conclusions

In this work, we developed novel Ru-containing catalysts supported by hyperbranched PPP and studied them in hydrogenation of LA to GVL. The decomposition of Ru acetylacetonate leads to the formation 2.7 nm Ru NPs with narrow NP size distribution. XPS revealed the presence of RuO_2_ species on the Ru^0^ NP core surface. The acidic modification of Ru-PPP leads to protonation of pyridine moieties and the incorporation of HSO_4_^−^ ions in the catalyst. The acidic functionalization of the catalyst (Ru-PPP-S) significantly increased the catalytic reaction rate and allowed for milder reaction conditions with quantitative GVL yields. This can be explained by several factors. First, protonated pyridine groups may enhance the catalytic activity due to ion pair formation with the substrate molecules acting as acidic co-catalyst. Second, hyperbranched PPP molecules may have a scavenging effect protecting Ru NPs from deactivation by sulfuric species. Finally, protons promoting the catalytic reaction do not leave the catalyst space due to hyperbranched character of the polymer. These results demonstrate that PPP is an excellent support in the Ru-catalyzed GVL production.

## Figures and Tables

**Table 1:** The results of LA hydrogenation in the presence of the Ru-PPP and Ru-PPP-S catalysts.

Catalyst	Reaction Conditions	LA Conversion (%)	GVL Yield (%)
Ru-PPP	100 °C, 2 MPa, 4 h	32.0	32
Ru-PPP	120 °C, 2 MPa, 4 h	68.2	68.2
Ru-PPP	130 °C, 2 MPa, 4 h	74.5	73.8
Ru-PPP	150 °C, 2 MPa, 4 h	83.5	81.8
Ru-PPP	100 °C, 3 MPa, 4 h	61.0	61.0
Ru-PPP	100 °C, 5 MPa, 4 h	77.5	77.5
Ru-PPP-S	100 °C, 2 MPa, 3 h 4 h	94.8 99.9	94.8 99.9
Ru-PPP-S	120 °C, 2 MPa, 3 h	98.9	98.9
Ru-PPP-S	130 °C, 2 MPa, 2 h 3 h	96.8 100	94.9 100
Ru-PPP-S	150 °C, 2 MPa, 1.5 h 2 h	94.8 99.9	92.9 98.9
Ru-PPP-S	100 °C, 3 MPa, 3 h	98.8	98.8
Ru-PPP-S	100 °C, 5 MPa, 2 h 3 h	95.4 100	94.4 99.8

**Table 2:** Comparison of different heterogeneous catalysts in the LA hydrogenation.

Catalyst	Solvent	Reaction Conditions	LA Conversion (%)	GVL Yield (%)	Ref.
5% Ru/C	dioxane	265 °C, H_2_ 1 bar, 1 g catalyst, 50 h	100	98.6	[29]
1% Ru/TiO_2_	H_2_O	70 °C, H_2_ 5 MPa, 0.3 g catalyst, 1 h	99	95	[26]
1% Ru/TiO_2_	H_2_O	150 °C, H_2_ 3.2 MPa, 0.4 mol% catalyst, 5 h	100	93	[30]
0.5% Ru/SiO_2_	H_2_O	130 °C, H_2_ 4 MPa, 0.1 g catalyst, 3 h	80	79	[28]
Ru/SiO_2_	H_2_O	90 °C, H_2_ 4.5 MPa, 0.4 mol% catalyst, 6 h	26	14	[57]
Cu-Al	H_2_O	200 °C, H_2_ 6 MPa, 0.2 g catalyst, 10 h	98	95	[58]
1% Pt/TiO_2_	GVL	200 °C, H_2_ 4 MPa, 1 wt% catalyst, 100 h	98	93	[59]
Ru_40_-DENs	H_2_O	150 °C, H_2_ 1 MPa, 0.5 mol% Ru, 5 h	100	99	[16]
Ru_40_@Meso-SiO_2_	H_2_O	150 °C, H_2_ 1 MPa, 0.5 mol% Ru, 5 h	94	94	[16]
Ru_40_@Meso-TiO_2_	H_2_O	150 °C, H_2_ 1 MPa, 0.5 mol% Ru, 5 h	92	90	[16]
1% Ru/zeolite-β	2-ethyl-hexanoic acid	200 °C, H_2_ 4 MPa, 0.3 g catalyst, 4 h	100	88	[27]
1% Ru/ZSM-5	2-ethyl-hexanoic acid	200 °C, H_2_ 4 MPa, 0.3 g catalyst, 4 h	100	90	[27]
1% Ru/Nb_2_O_5_	2-ethyl-hexanoic acid	200 °C, H_2_ 4 MPa, 0.3 g catalyst, 5 h	95	93	[27]
5% Ru/SiO_2_	H_2_O	70 °C, H_2_ 0.5 MPa, 0.5 mol% Ru, 4 h	88	84	[60]
5% Ru/ZrO_2_	H_2_O	70 °C, H_2_ 0.5 MPa, 0.5 mol% Ru, 4 h	69	67	[60]
5% Ru/MCM-41	H_2_O	70 °C, H_2_ 0.5 MPa, 0.5 mol% Ru, 4 h	89	84	[60]
1% Ru/OMC/H_3_PO_4_	H_2_O	70 °C, H_2_ 0.7 MPa, 0.1 mol% Ru, 6 h	98	92	[22]
1% Ru/OMC/H_3_PO_4_	H_2_O	200 °C, H_2_ 4 MPa, 0.1 mol% Ru, 6 h	99	67	[22]
3.5% G2-dendr-SiO_2_-Ru	H_2_O	120 °C, H_2_ 3 MPa, 2 h	84	78	[14]
Ru-PPP-S	H_2_O	100 °C, 2 MPa, 0.016 mol% Ru, 3 h 4 h	94.8 99.9	94.8 99.9	this work

## Data Availability

The data presented in this study are available upon request from the corresponding author.

## Supplementary Materials

The following supporting information can be downloaded at: https://www.mdpi.com/article/10.3390/ijms23020799/s1. References [44,63] are cited in Supplementary Materials.
