# Supplementary material for: Ru@hyperbranched Polymer for Hydrogenation of Levulinic Acid to Gamma-Valerolactone: The Role of the Catalyst Support
Source: Int J Mol Sci. 2022 Jan 12;23(2):799. doi: 10.3390/ijms23020799 (PMC8776037; doi:10.3390/ijms23020799)
Supplement: Supplementary file 1 [file ijms-23-00799-s001.zip › ijms-1498167-supplementary.pdf]

Supplementary Material

Ru@hyperbranched polymer for hydrogenation of levulinic acid to gamma-valerolactone: The role of the catalyst support

FTIR data

Several bands at 840 – 610 cm^{-1} are assigned to out-of-plane deformation vibrations of C-H in phenylene and pyridine rings of PPP. The bands at 1225-950 cm^{-1} in Ru-PPP are attributed to substituent sensitive in-plane deformation vibrations of C-H of aromatic rings. The broad peaks at 1614 - 1400 cm^{-1} are characteristic for stretching of aromatic rings, while the signal at 1695 cm^{-1} is attributed to C=N ring stretching vibrations for both samples. Several lines at 3100-3000 cm^{-1} correspond to C-H stretching vibrations of substituent pyridine, while bands at 2900-2800 cm^{-1} present the stretching of phenylene C-H groups. Bands at 2950-2900 cm^{-1} are typically seen in sulfonic acid spectrum because of OH stretching, while the signals at 2868-2838 cm^{-1} have arisen due to overtones or combinations of hydrogen bonded OH bending vibration.[1]

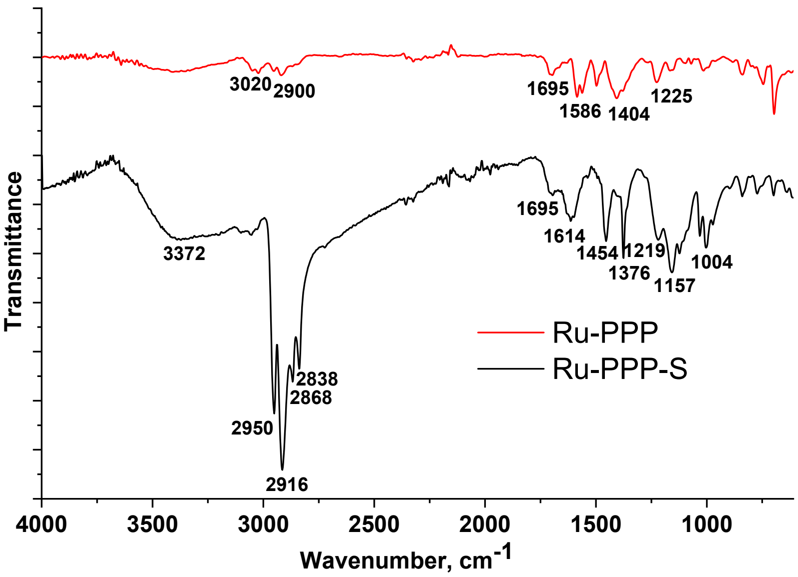

Figure S1. FTIR spectra of Ru-PPP (red line) and Ru-PPP-S (black line).

XRD

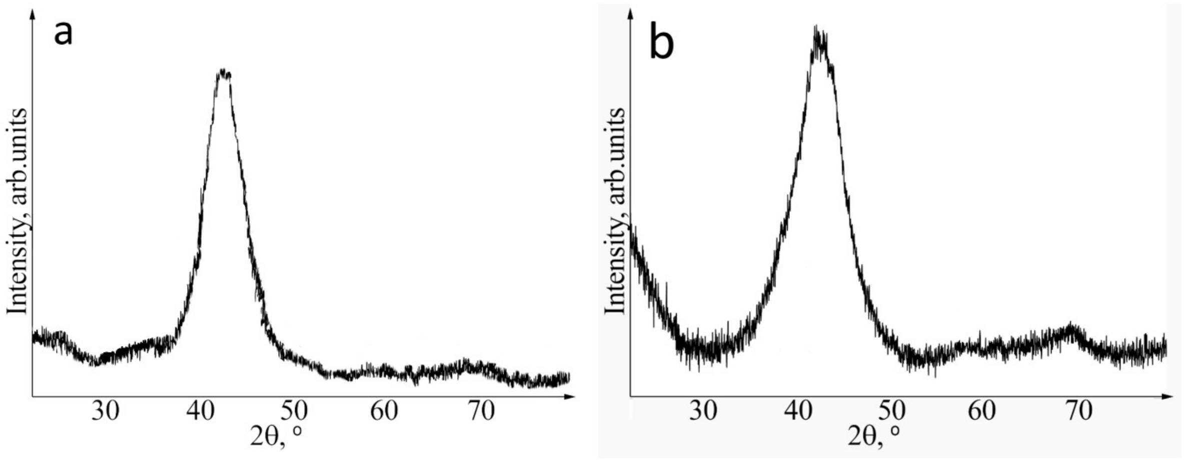

Figure S2. XRD pattern of Ru-PPP (a) and Ru-PPP-S (b)

XPS

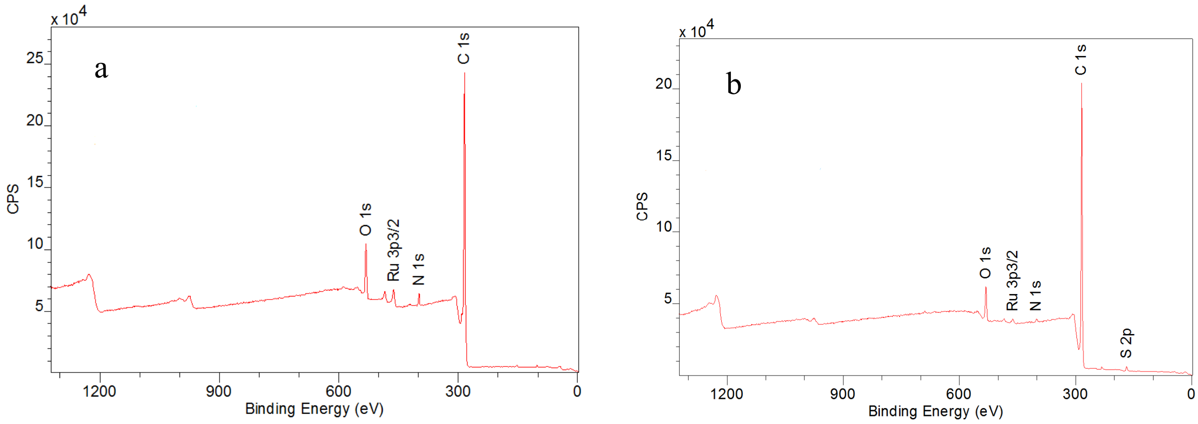

Figure S3. XPS survey spectra of Ru-PPP (a) and Ru-PPP-S (b).

Table S1. The elemental composition of Ru-PPP and Ru-PPP-S from XPS.

Name	Element	%at	%wt
Ru-PPP	O 1s	5.66	6.89
	Ru $3p_{3/2}$	0.91	7.00
	N 1s	1.54	1.64
	C 1s	91.89	84.46
Ru-PPP-S	C 1s	93.15	88.66
	O 1s	4.75	5.93
	N 1s	0.74	0.81
	S 2p	1.14	2.85
	Ru $3p_{3/2}$	0.22	1.76

The high resolution (HR) XPS C 1s spectrum has been deconvoluted into several peaks assigned to sp^2 carbon of aromatic rings of PPP, C-N carbon of pyridine moieties, C-O presented in ether groups of PPP, π-π interactions in sp^2 carbon and C 1s of COOH groups which are most probably the result of oxidation of residual ethynyl groups of PPP in the course of high temperature synthesis of Ru NPs. The deconvolution parameters are presented in Table S3 and S4 consistent with literature data [2].

Table S2. Fitting parameters for HR XPS C1s of Ru-PPP and Ru-PPP-S.

Sample name	Band	Position, eV	FWHM, eV	%Area
Ru-PPP	1	284.67	1.15	60249
	2	285.3	1.26	26311
	3	286.27	1.54	8855
	4	291.65	2.5	5374
	5	288.81	2.5	3499
Ru-PPP-S	1	284.76	1.08	65189
	2	285.33	1.14	9542
	3	286.2	1.78	5431
	4	291.37	1.21	128
	5	288.69	1.67	617

Table S3. Fitting parameters for HR XPS Ru 3d of Ru-PPP and Ru-PPP-S

Sample name	Band	Position, eV	FWHM, eV	%Area
Ru-PPP	1	281.16	1.32	10103
	2	285.33	1.59	6746
	3	283.06	1.32	5001
	4	287.43	1.59	3339
	5	280.10	1.0	4890
	6	284.27	1.1	3260
Ru-PPP-S	1	280.81	1.33	3967
	2	284.98	1.60	2649
	3	282.71	1.33	1963
	4	287.08	160	1311
	5	279.70	1.60	333
	6	283.87	1.76	222

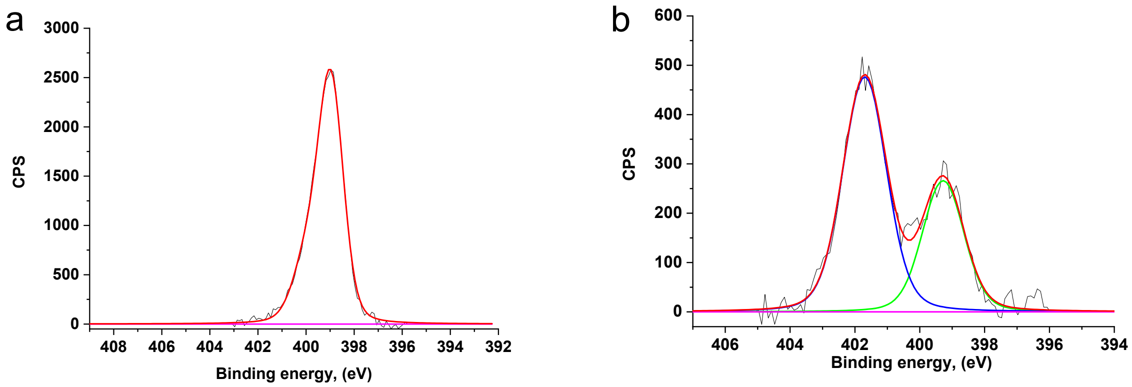

Figure S4. HR XPS N 1s of Ru-PPP (a) and Ru-PPP-S (b). Black line is the experimental data, the blue line is for H-N^+ (in pyridinium moieties), the green line is for N (in pyridine moieties), the magenta line is for background and the red line is for the fitting curve.

Table S4. Fitting parameters for HR XPS N 1s of Ru-PPP-S.

Band	Position, eV	FWHM, eV	%Area
1	399.28	1.51	462
2	401.70	1.59	875

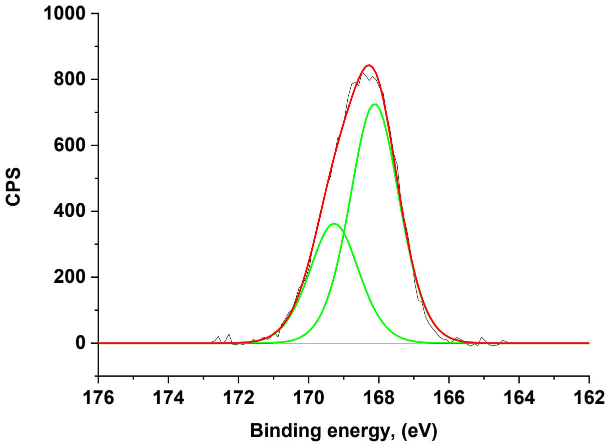

Figure S5. HR XPS S 2p of Ru-PPP-S. Black line is the experimental data, the green lines are for S 2p 3/2 and S 2p ½, the blue line is for background and the red line is for the fitting curve.

Table S5. Fitting parameters for HR XPS S 2p of Ru-PPP-S.

Band	Position, eV	FWHM, eV	%Area
1	168.12	1.66	1317
2	169.27	1.66	658

TGA

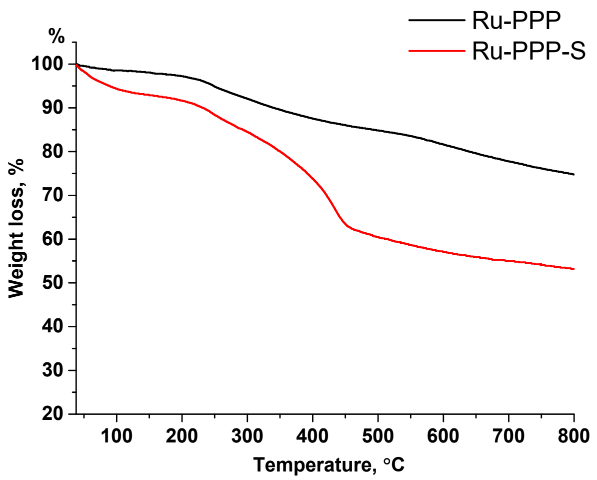

Figure S6. TGA profiles for Ru-PPP (black line) and Ru-PPP-S (red line) in argon atmosphere.

References

1. Panicker, C.Y.; Varghese, H.T.; Philip, D.; Nogueira, H.I. FT-IR, FT-Raman and SERS spectra of pyridine-3-sulfonic acid. *Spectrochimica acta. Part A, Molecular and biomolecular spectroscopy* **2006**, *64*, 744-747, doi:10.1016/j.saa.2005.06.048.
2. Wagner, C.D.; Rigs, W.M. *Handbook of X-ray photoelectron spectroscopy. NIST X-ray Photoelectron Spectroscopy Database*; Perkin-Elmer Corporation: 1979.
